# S2k-Guideline SARS-CoV-2, COVID-19 and (early) rehabilitation – a consensus-based guideline for Germany

**DOI:** 10.3205/dgkh000438

**Published:** 2023-05-12

**Authors:** Thomas Platz, Peter Berlit, Christian Dohle, Helmut Fickenscher, Manju Guha, Volker Köllner, Axel Kramer, Rembert Koczulla, Axel Schlitt

**Affiliations:** 1 BDH-Klinik Greifswald, Greifswald, Germany; 2 Neurorehabilitation Research Group, Universitätsmedizin Greifswald, Greifswald, Germany; 3 German Society of Neurology Berlin, Berlin, Germany; 4 P.A.N. Center for Post-Acute Neurorehabilitation, Fürst-Donnersmarck-Stiftung, Berlin, Germany; 5 Center for Stroke Research Berlin, Charité – University Berlin, Berlin, Germany; 6 Institute for Infection Medicine, Kiel University and University Medical Center Schlewsig-Holstein, Kiel, Germany; 7 Reha-Klinik am Sendesaal Bremen, Abteilung Kardiologie, Bremen, Germany; 8 Department of Psychosomatic Medicine, Rehabilitation Center Seehof, Federal German Pension Agency, and Research Group Psychosomatic Rehabilitation, Charité University Medicine Berlin, Teltow/Berlin, Germany; 9 Institute of Hygiene and Environmental Medicine, University Medicine Greifswald, Greifswald, Germany; 10 Institute for Pulmonary Rehabilitation Research, Schön Klinik Berchtesgadener Land, Schönau am Königssee, Germany; 11 Department of Pulmonary Rehabilitation, Member of the German Center for Lung Research (DZL), University Medical Center Giessen and Marburg, Philipps University Marburg (UGMLC), Marburg, Germany; 12 Teaching Hospital, Paracelsus Medical University Salzburg, Salzburg, Austria; 13 Paracelsus-Harz Clinic Bad Suderode, Quedlinburg, Germany; 14 Medical Faculty, Martin-Luther University Halle-Wittenberg, Germany

**Keywords:** COVID-19, Post COVID, practice recommendation, consensus based guideline, intensive care, rehabilitation, training, paresis, cognition, fatigue, emotion

## Abstract

The consensus-based guideline “SARS-CoV-2, COVID-19 and (early) rehabilitation” for Germany has two sections: In the first part, the guideline addresses infection protection-related procedures during the COVID-19 pandemic. In the second part, it provides practice recommendations for rehabilitation after COVID-19.

The specific recommendations for rehabilitation after COVID-19 as issued by 13 German medical societies and two patient-representative organizations are presented together with general background information for their development.

## Abstract (de)

Die Leitlinie „SARS-CoV-2, COVID-19 und (Früh-)Rehabilitation“ wurde nach dem Ausbruch der COVID-19 Pandemie von 13 medizinischen Fachgesellschaften und zwei Patientenvertretungen als konsensbasierte Arbeitsgrundlage einerseits zur Prävention von SARS-CoV-2 Infektionen in Einrichtungen der (Früh-)Rehabilitation für Deutschland erarbeitet, andererseits, um spezifische Empfehlungen für die (Früh-)Rehabilitation von COVID-19-Betroffenen fachgesellschaftsübergreifend zu geben.

Im ersten Teil befasst sich die Leitlinie daher mit infektionsschutzrelevanten Maßnahmen während der COVID-19-Pandemie einschließlich der den Empfehlungen zugrunde liegenden Evidenz. Im zweiten Teil werden Praxisempfehlungen für die Rehabilitation nach COVID-19 gegeben. Die englischsprachige Kurzfassung gibt die Empfehlungen dieses zweiten Teils mit kurzgefassten Hintergrundsinformationen zu ihrer Entwicklung wieder.

## Guideline

Guideline of the German Society for Neurorehabilitation in cooperation with the German Society for Hematology and Medical Oncology, German Society for Hygiene and Microbiology, German Society for Cardiology, Heart and Circulation Research, German Society of Pediatrics and Adolescent Medicine, German Society for Clinical Psychotherapy, Prevention and Psychosomatic Rehabilitation, German Society of Hospital Hygiene, German Neurological Society, German Society of Physical and Rehabilitation Medicine, German Society for Pneumology and Respiratory Medicine, German Society for Prevention and Rehabilitation of Cardiovascular Disease, German Society for Rehabilitation Sciences, BDH Federal Association Rehabilitation and Long COVID Germany

## Overview

The original guideline (in German), first published in 2020, has two sections: In the first part, the guideline addresses the infection protection-related procedures during the COVID-19 pandemic and in its transition to endemic. In the second part, it describes the care of COVID-19 patients with rehabilitative measures from the intensive care unit and acute-care hospitalisation, to early rehabilitation, post-acute rehabilitation, outpatient, and long-term care after COVID-19, with a focus on prolonged persistence of symptoms (Long COVID or Post COVID).

As we are in the transition from a pandemic to an endemic situation, this abbreviated English version of the guideline will present the practice recommendations for rehabilitation after COVID-19 (only). It will be of interest for an international readership to become acquainted with the specific recommendations issued by medical societies and patient-representative organisations for rehabilitation after COVID-19. 

## Guideline development process

The first version of the guideline was published in November 2020, then subsequently the 1^st^ update in November 2021, and is now available as the updated version 3 (2^nd^ update) (date: November 1^st^, 2022) [1].

Delegates of thirteen medical societies from various medical fields, including cardiology, haematology/oncology, hygiene, pulmonology, neurology, paediatrics, physical medicine and rehabilitation, psychosomatic medicine, and virology, as well as two patient representatives’ organizations jointly developed the guideline. 

The process included a critical appraisal of the currently available evidence, drafting the guideline manuscript with chapters being allocated to author groups, and deducing recommendations as a draft. Once the first version of the (updated) guideline was available, it was critically reviewed by all authors of the guideline development group, and revised accordingly. Afterwards, the draft was sent out to the delegates for corona virus disease 2019 (COVID-19) and related questions of many national medical societies acting together as “Covid-19 Task force” of the Association of the Scientific Medical Societies in Germany (AWMF) for their review. Their comments were integrated. As the next step, a formal consensus conference was held. All recommendations were adopted in a structured consensus process in October 2022 with neutral moderation by the AWMF among the mandate holders of the professional societies involved (guideline development group). 

As this guideline was updated a second time, any previously issued recommendations were “reviewed” and could be considered as still valid in the course of updating the guideline without a need for modification, or could be “modified” during the review. In addition, “new” recommendations were included in the current version of the guideline. The degree of consensus was defined as either “strong consensus“ (>95% of participants agree), “consensus” (>75–95% of participants agree), “majority agreement” (>50–75% of participants agree), or “no majority agreement” (≤50% of participants agree). The information regarding the recommendation’s history (i.e. “reviewed”, “modified”, or “new”) and the degree of consensus achieved are provided for each recommendation (in brackets).

After the consensus conference, the final version of the guideline was sent out to the office of each of the thirteen participating medical societies and the two patient representatives’ organizations. After approval of the final document by all participating societies, the guideline was published online. 

The purpose of this abbreviated publication is mainly to present the consensus-based practice recommendations issued to a broader international readership. For detailed background information and references related to the recommendations provided below, the guideline text should be consulted [1].

## Assessment of the necessity for and extent of rehabilitative measures for people affected by COVID-19

In the rehabilitation context, it is important to individually determine any COVID-19-associated organ, physical dysfunctions, or emotional disorders and characterise their relevance for daily life by use of appropriate assessments. Based on such individualised diagnostics and assessments, the need for rehabilitative measures – also in the longer term – can be individually identified medically and psychosocially.

The guideline follows a pragmatic nomenclature of post-COVID sequelae that may be documented soon (from 4 weeks) after COVID-19 (“Long COVID”) or after a longer period of time (from 12 weeks; “Post-COVID Syndrome, PCS”).

Long- or post-COVID Syndrome (PCS) must be distinguished from both other (organic) diseases and from stress and associated health disorders which are related to the effects of the pandemic on personal life (but not caused by a SARS-CoV-2 infection) [2].

Although this pragmatic approach with a distinction between long-COVID and PCS is chosen, the guideline makes it clear that there are different groups of people with long-COVID and PCS. This is due to the fact that COVID-19 can cause very diverse organ damage, e.g. to the lungs, cardiovascular system, central nervous system, peripheral nervous system, liver, kidneys and/or muscles, as well as emotional disorders that can result in functional restrictions that may persist and be detrimental for everyday life and work. Such organ damage and any resulting impairments and activity limitations result in very different individual rehabilitation needs, both with regard to medical care (also in combination) and the duration of necessary rehabilitation measures.

The different medical needs for rehabilitation that may be occur in people affected by long Covid/PCS, e.g. in the pneumological, neurological, cardiological and/or psychosomatic fields, have been made transparent in the guideline, based on the currently available evidence.

Furthermore, also within a medical specialty focus for rehabilitation among long COVID and PCS, further patient subgroups with different care needs can be differentiated. For example, for the clinical management of individuals suffering from long Covid/PCS with predominantly neurological complaints (including cognition), two patient subgroups can be distinguished: Group A, patients with neurological physical dysfunctions after a severe to critical course of COVID-19 that persist beyond the acute phase (who may have “post intensive care syndrome, PICS” with motor, cognitive and emotional disturbances), and Group B, patients who – after a primarily mild and moderate course of COVID-19 – may only suffer from neurological functional disorders at a later time point and exhibit a different pattern of symptoms that nevertheless restricts participation in their social and working life. This distinction of subgroups was statistically supported by cluster analysis [3].

Another example for such inter-individual diversity is the fatigue syndrome, which is reported by a considerable percentage of non-hospitalized COVID-19 sufferers [4] as subjectively severely limiting exhaustion on a somatic, cognitive and/or psychological level, disproportionate to the foregoing activities/efforts and not sufficiently improving through sleep or rest. Fatigue after COVID-19 frequently improves over the course of the first 6 months after COVID-19 and to a greater extent than the cognitive impairments that are frequently mentioned [5]. On the other hand, a fatigue syndrome can also persist for a long time and be associated with strong and prolonged exercise intolerance (postexertional malaise, PEM) [6]. In the case of severe and particularly long-lasting PEM (≥14 hours) that continues for at least 6 months, chronic fatigue syndrome (postviral fatigue syndrome, myalgic encephalomyelitis), ME/CFS, can be diagnosed when the other criteria for this syndrome are also present. Importantly, in this small subgroup of people affected by post-COVID with severe and prolonged exercise intolerance, a specific need exists for treatment, since training therapy, which is often a priority in medical rehabilitation, is frequently not effective for these individuals or can even lead to an increase in symptoms and clinical deterioration [7]. For the vast majority of patients, individually-tailored exercise therapy, teaching them to recognize and respect their own exercise limits, is effective and safe. It is important to educate patients about this, as dysfunctional fears of physical activity can worsen quality of life.

Another problem is the tendency to pathological self-exhaustion that perpetuates fatigue symptomatology. The diagnosis and treatment require close communication between the professional groups involved. A theoretical framework is provided, for example, by the avoidance/endurance concept of pain chronification, which focuses on both a dysfunctional tendency to overtax oneself and a protective behavior that perpetuates the symptomatology and can be transferred well to fatigue symptomatology. In the meantime, first proofs of effectiveness for an interdisciplinary rehabilitation on this basis are available [8].

In quite a few PCS sufferers (e.g., in the case of an initial course of the disease requiring intensive care or in the case of strong and prolonged stress intolerance), longer-term restrictions relevant for their employment can be observed [9], which then require specific “return to work” support and professional integration management to promote participation.

These examples make it clear that within the terminology of long COVID and PCS, very different case constellations are possible, which thus require individualized diagnostic measures and, based on these, an individualized and often interdisciplinary rehabilitation plan. This guideline intends to make these complexes transparent and hence promote a deeper understanding of possible constellations within the umbrella terms long COVID and PCS and any individual rehabilitative care necessary.

## Recommendations for rehabilitation after COVID-19

1. Rehabilitative treatment approaches should already be carried out in the intensive care unit and, if necessary, continue with interdisciplinary early rehabilitation in the acute hospital (reviewed; strong consensus).
2. In the case of pulmonary weaning failure, long COVID/PCS sufferers should be cared for in a pulmonologist-led or anesthesiologist-led weaning unit for prolonged weaning from a ventilator (reviewed; strong consensus).
3. (COVID-19 and) long COVID/PCS patients with relevant disorders of the peripheral and/or the central nervous system should undergo early neurological rehabilitation, this individually also includes prolonged weaning from a ventilator (reviewed; strong consensus).
4. Before a long COVID/PCS patient on a ventilator is discharged into out-of-hospital intensive nursing care, the potential to wean from ventilation should be checked by qualified physicians (reviewed; strong consensus).
5. Especially in patients after severe and critical courses, symptoms (e.g., dyspnea on exertion, poor performance), organ damage (e.g., lungs, cardiovascular system, CNS, PNS, liver, kidneys and muscles) as well as psychological disturbances persist at relatively high rates once they have survived the acute phase. In such cases, rehabilitation should be initiated, usually initially as in-patient rehabilitation (reviewed; strong consensus).
6. If, for example, the pulmonary, cardiac, or neurological damage (impairment) leads to the need for rehabilitation, a specific pneumological, cardiological, or neurological in-patient or out-patient rehabilitation should be undertaken (reviewed; strong consensus).
7. If there is a pronounced exercise intolerance in the context of postviral fatigue after COVID-19, a specialized treatment concept should be offered (new; strong consensus).
8. Due to the frequency of psychological consequences of a SARS-CoV-2 infection (and the often pronounced avoidance of those affected to spontaneously report about them), systematic screening with suitable questions or short questionnaires to record psychological consequences should be carried out. This should already be done during acute treatment in the hospital in order to enable professional psychosomatic/psychotherapeutic treatment in the acute-care hospital or to facilitate it in the rehabilitation hospital (modified; strong consensus).
9. If necessary, there should be intensive psychosomatic/psychiatric/psychological support for those affected, e.g., for the following aspects: dealing with general, illness-related and post-traumatic fears and depression, experiences of isolation and quarantine, coping strategies for chronic symptoms, worries about the future and recovery of functioning (modified; strong consensus).
10. In the case of psychological consequences of a SARS-CoV-2 infection, the indication for psychosomatic (outpatient or) in-patient rehabilitation should be checked in the case of persistent or exacerbating symptoms during out-patient treatment (reviewed; strong consensus).
11. During rehabilitation – based on the socio-medical assessment – further steps of medical, vocational or social rehabilitation should also be initiated (reviewed; strong consensus).
12. If a primary need for rehabilitation existed after the acute illness phase, the progress of rehabilitation and any further need for rehabilitation, therapy or psychosocial support should be checked at least quarterly in the first year after the acute illness (reviewed; strong consensus).
13. For the treatment of long COVID/PCS-related restrictions (in people with an initially mild COVID-19 course), the medical diagnostic clarification should be followed by prescription of therapy and treatments primarily as out-patient care in order to restore the impaired body functions, and counteract activity limitations and the resulting restrictions in participation. In particular, these include out-patient physiotherapy, physical therapy, sports and exercise therapy, occupational therapy, neuropsychology and/or speech therapy. If indicated, psychotherapy should be initiated (modified; strong consensus).
14. Out-patient or in-patient medical rehabilitation should be prescribed for long COVID/PCS sufferers if, after COVID-19, there are not only temporary restrictions of participation in community life (or a threat thereof) that require multimodal medical and therapeutic treatment, i.e., if out-patient measures are not sufficient for treatment (reviewed; strong consensus).
15. In the case of chronic functional limitations (including cognition) in PCS sufferers of working age, the indication for measures to participate in working life (“return to work”) or vocational integration management should be checked and appropriate measures should be initiated in addition to medical rehabilitation (new; strong consensus).
16. People who contracted COVID-19 for work-related reasons, suffer from the consequences, and want a medical check-up for long-/post-COVID can contact their social security agency for occupational diseases; the corresponding contact information should be made available to them (reviewed; strong consensus).

## Conclusion

Among those who suffer from long COVID and PCS, very different constellations of impairments of body functions and concomitant activity limitations as well as restricted participation are possible. This is because long COVID and PCS are “umbrella” terms, based on the temporal evolution of symptoms, while COVID-19 may lead to diverse organ dysfunctions and cognitive and emotional disturbances (and their combinations), depending on the individual. Accordingly, an individualized diagnostic approach is necessary and based on that, an individualized rehabilitation plan makes sense (if indicated). Rehabilitation measures as individually required, including those on the intensive care unit, might be necessary for early or post-acute in-patient rehabilitation, out-patient treatment or multi-professional rehabilitation at a later stage, and – depending on the individual case – may have a pneumological, neurological, cardiological, or psychosomatic focus. In many cases, an interdisciplinary approach is necessary. Frequently, the individual diagnostic work-up and treatment including rehabilitation will be based on a multi- and inter-professional approach.

## Participating organizations

### Scientific medical societies

**Deutsche Gesellschaft für Neurorehabilitation (DGNR) e.V.**; elected official Prof. Dr. med. Thomas Platz, Greifswald; (elected official (substitute): PD Dr. med. Christian Dohle) 

**Deutsche Gesellschaft für Hämatologie und Medizinische Onkologie (DGHO) e.V.**; elected official Dr. med. Monika Steimann (elected official (substitute): Dr. med. Imke Strohscheer) 

**Deutsche Gesellschaft für Hygiene und Mikrobiologie (DGHM) e.V.**; elected official Prof. Dr. med. Helmut Fickenscher 

**Deutsche Gesellschaft für Kardiologie – Herz- und Kreislaufforschung (DGK) e.V.**; elected official Dr. med. Manju Guha (elected official (substitute): apl.-Prof. Dr. med. Axel Schlitt) 

**Deutsche Gesellschaft für Kinder- und Jugendmedizin (DGKJ) e.V.**; elected official Dr. med. Stefan Berghem

**Deutsche Gesellschaft für Klinische Psychotherapie, Prävention und Psychosomatische Rehabilitation (DGPPR) e.V.**; elected official Prof. Dr. med. Volker Köllner (elected official (substitute): Prof. Dr. med. Markus Bassler) 

**Deutsche Gesellschaft für Krankenhaushygiene (DGKH) e.V.**; elected official Prof. Dr. med. Axel Kramer 

**Deutsche Gesellschaft für Neurologie (DGN) e.V.**; elected official Prof. Dr. med. Peter Berlit 

**Deutsche Gesellschaft für Physikalische und Rehabilitative Medizin (DGPRM) e.V.**; elected official Dr. med. Annett Reißhauer (elected official (substitute): Dr. med. Maximilian Liebl) 

**Deutsche Gesellschaft für Pneumologie und Beatmungsmedizin (DGP) e.V.**; elected official Dr. med. Stefan Dewey (elected official (substitute): Prof. Dr. med. Michael Pfeifer) 

**Deutsche Gesellschaft für Prävention und Rehabilitation von Herz-Kreislauferkrankungen (DGPR) e.V.**; elected official apl.-Prof. Dr. med. Axel Schlitt (elected official (substitute): Dr. med. Manju Guha) 

**Deutsche Gesellschaft für Rehabilitationswissenschaften (DGRW) e.V.**; elected official Prof. Dr. med. A. Rembert Koczulla (elected official (substitute): apl.-Prof. Dr. med. Axel Schlitt) 

**Gesellschaft für Virologie (GfV) e. V.**; elected official Prof. Dr. med. Helmut Fickenscher 

### Patient-representative organizations

**BDH Bundesverband Rehabilitation (BDH) e.V. (BDH)**; elected official Ulrike Abel 

**Betroffenen-Initiative Long COVID Deutschland (LCD)**; elected official Dr. med. Claudia Ellert (elected official (substitute): Nadine Rommerswinkel) 

### Organization without voting rights

**Deutsche Vereinigung zur Bekämpfung der Viruskrankheiten (DVV) e.V.**; elected official Prof. Dr. med. Helmut Fickenscher 

## Notes

### Competing interests

The authors declare that they have no competing interests.

### Acknowledgments

This work was supported by the BDH Bundesverband Rehabilitation e.V. (charity for neuro-disabilities) by a non-restricted personal grant to TP. The sponsors had no role in the decision to publish or the content of the publication. 

### Authors’ ORCIDs:

- Thomas Platz: 0000-0003-2629-9744
- Axel Kramer: 0000-0003-4193-2149
